# Augmented reality enhanced physical simulation for femoral nailing training

**DOI:** 10.1007/s11548-026-03646-8

**Published:** 2026-06-06

**Authors:** Tobias Stauffer, Blanca Gutiez, Reto Babst, Quentin Lohmeyer, Mirko Meboldt

**Affiliations:** 1 Department of Mechanical and Process Engineering, ETH Zürich, Rämistrasse 101, 8092 Zürich, Switzerland; 2 Department of Orthopedics and Trauma Surgery, Lucerne Cantonal Hospital, Spitalstrasse 34, 6000 Lucerne, Switzerland; 3 Faculty of Health Science and Medicine, University Lucerne, Alpenquai 4, 6005 Lucerne, Switzerland

**Keywords:** Augmented Reality (AR), Surgical Training Simulation, Femoral Nailing, Fluoroscopic Navigation

## Abstract

**Purpose:**

Current femoral nailing simulators face a trade-off between delivering procedural guidance and realistic haptic feedback. This study aimed to address this limitation by developing and evaluating an augmented reality (AR)-enhanced physical simulator that combines real surgical instruments with AR-based visualization. The system provides spatial feedback on fluoroscopic imaging orientation and implant placement within hidden anatomical structures, supporting self-directed learning.

**Methods:**

A hybrid simulator integrating a synthetic femur, real surgical instruments, and an AR interface on the Apple Vision Pro was developed. The AR application introduces users to fluoroscopic imaging and implant placement—covering nail insertion and alignment. After completing this AR-guided phase, 25 participants (6 experts and 19 non-experts) performed three unguided simulation runs followed by one retention session. Performance metrics included task completion time, number of simulated fluoroscopic images, and implant placement accuracy.

**Results:**

Data from 23 participants were analyzed. Task completion time decreased across simulation runs (insertion: −47.1%; alignment: −57.4%) and remained stable during retention. The number of simulated fluoroscopic images declined (insertion: −45.6%; alignment: −49.2%). Implant placement accuracy remained stable. Experts consistently performed faster than non-experts, with performance differences decreasing across simulation runs.

**Conclusion:**

The AR-enhanced physical simulator led to clear learning effects in procedural efficiency and radiographic handling while maintaining consistent implant accuracy. These findings support AR as a valuable tool to enhance accessibility, spatial learning, and realism in simulation-based surgical training, representing a further step in the ongoing shift toward simulation-based surgical training.

## Introduction

Surgery training is currently undergoing a shift from tutor-based training on cadavers or patients to digital, simulation-based training [1, 2]. This transition allows trainees improved access, enables repetitive practice, and provides objective assessment methods—all without putting patients at risk [3, 4]. Numerous simulators have been developed for intramedullary nailing, a widely performed procedure for the treatment of proximal femur and femoral shaft fractures, which are among the most common long-bone fractures, with reported incidences on the order of 10–30 cases per 100,000 people per year in population-based studies [5, 6]. Mastering this operation requires a strong three-dimensional understanding of patient anatomy and strong motor skills as the procedure relies heavily on fluoroscopic imaging for navigation [7, 8].

Many of the developed simulators are based on virtual reality [9–11]. With these systems, entire procedures can be practiced independently. Studies have shown that these simulators are promising for learning the surgical technique, especially in becoming familiar with the procedural steps [9, 12, 13]. However, due to the lack of haptic feedback, the acquisition of motor skills is limited, which impedes the overall learning progression [14, 15]. In addition to virtual systems, a few femoral nailing simulators have been developed that employ physical setups to provide realistic haptic feedback [16, 17]. By combining instrument motion tracking with simulated fluoroscopic imaging, these simulators allow trainees to practice critical procedural steps and concurrently develop both motor skills and fluoroscopic navigation competence. Studies have shown that such systems can effectively enhance trainee performance in both areas [16, 18]. However, compared to virtual reality simulators, they lack procedural guidance, making unsupervised training difficult and thereby reducing accessibility. To address these limitations, this work proposes a hybrid simulator that combines the realistic haptics of a physical setup with augmented reality (AR)-based procedural guidance, enabling self-directed and accessible training. For learning the femoral nailing procedure, AR offers two key advantages: (1) it provides spatial feedback on the orientation of simulated fluoroscopic imaging directly on the setup, helping trainees understand the three-dimensional relationship between the C-arm, the patient, and the implant; and (2) it visualizes hidden anatomy and implant position, giving intuitive feedback on procedural accuracy. These capabilities make AR particularly well suited to bridge the gap between purely virtual and purely physical simulators.

To assess the effectiveness of the proposed simulator, a study with 25 participants was conducted. Learning curves were evaluated across simulation and retention sessions based on two outcome categories: time on task and procedural quality reflected by the number of fluoroscopic images taken and the accuracy of implant placement. The results are expected to demonstrate the potential of AR in enhancing physical phantom setups with realistic haptics, enabling the learning of three-dimensional understanding of patient anatomy and motor skills, eliminating the need for a physically present instructor, which would represent another step forward in the ongoing shift toward simulation-based surgical training.

## Methods

### Physical setup

**Fig. 1:**
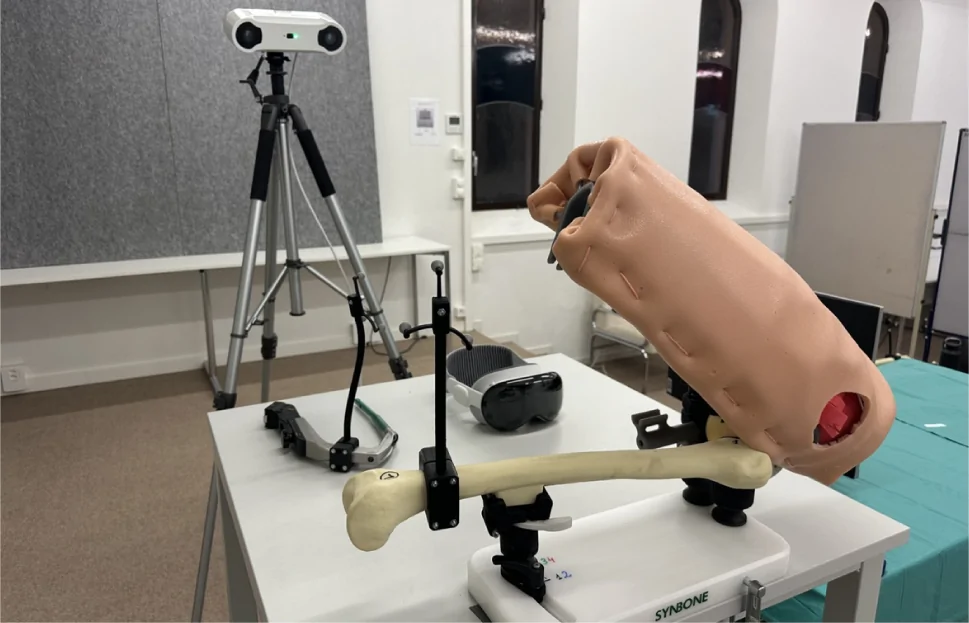
Physical femoral nailing simulator setup. A synthetic proximal femur covered by a soft tissue surrogate restricts visual and manual access, replicating the exposure conditions of minimally invasive femoral nailing. Real surgical instruments are used to provide realistic haptic feedback during implant manipulation

The training setup is shown in Fig. 1; it consists of a synthetic proximal femur (SYNBONE AG, Switzerland) mounted in an anatomically correct position using a bone holder. The model is enclosed in a soft tissue cover that limits visual and manual access to the femur, thereby reproducing the restricted exposure characteristic of femoral nailing procedures. Furthermore, the simulator includes a short femoral nail, an aiming arm, and a surgical hammer (all Auxein Medical Pvt. Ltd., India).

**Fig. 2:**
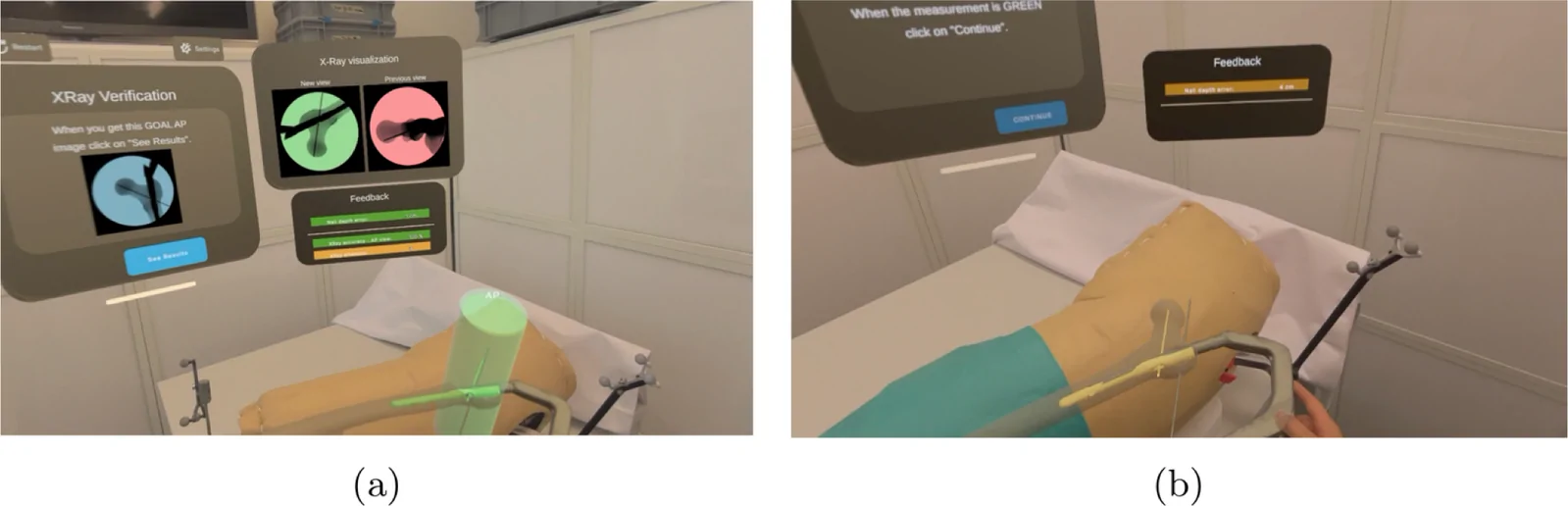
AR-based procedural guidance for the nail insertion step: **a** Instruction on simulated fluoroscopic imaging: Users are guided to align the virtual X-ray beam with the optimal projection direction to obtain the target view. **b** Instruction on accurate implant placement: The AR interface visualizes hidden anatomy and target implant position, providing real-time feedback on nail positioning

### AR application

The AR training application was developed in Unity (Unity Technologies, USA) and deployed on the Apple Vision Pro (Apple Inc., USA). As illustrated in Fig. 2, the application guides users through the insertion and alignment steps of a femoral nailing procedure, introducing two core procedural objectives: (1) use of simulated fluoroscopic imaging and (2) accurate placement of implants. Each task is introduced with a short instructional video displayed in the AR interface, demonstrating the corresponding surgical maneuver. Throughout the guidance, users receive multimodal instructions combining numerical indicators and color-coded 3D overlays. This design choice was intended to foster user confidence and engagement [19], while enhancing spatial understanding [20].

**Instruction on fluoroscopic imaging:** As illustrated in Fig. 2a, users are guided to obtain the fluoroscopic projections required for the two procedural steps defined in the training. An anchored virtual cylinder representing the X-ray beam volume is displayed in AR to indicate the optimal imaging orientation. Guided by color-coded overlays, users adjust a second cylinder’s position and angle until the target view—anteroposterior (AP) for insertion and axial for rotational alignment—is achieved. Once the orientation is correct, the indicator cylinder turns green to confirm the imaging direction, and a simulated X-ray can be taken.

**Instruction on implant placement:** As illustrated in Fig. 2b, users are guided to achieve the target insertion depth and rotational alignment of the femoral nail. The AR interface visualizes the hidden anatomy and the target implant position, providing real-time spatial feedback. Using color-coded overlays, users adjust insertion and rotation until the target position is achieved, at which point the indicator turns green to confirm accurate placement.

### Data processing

**Fig. 3:**
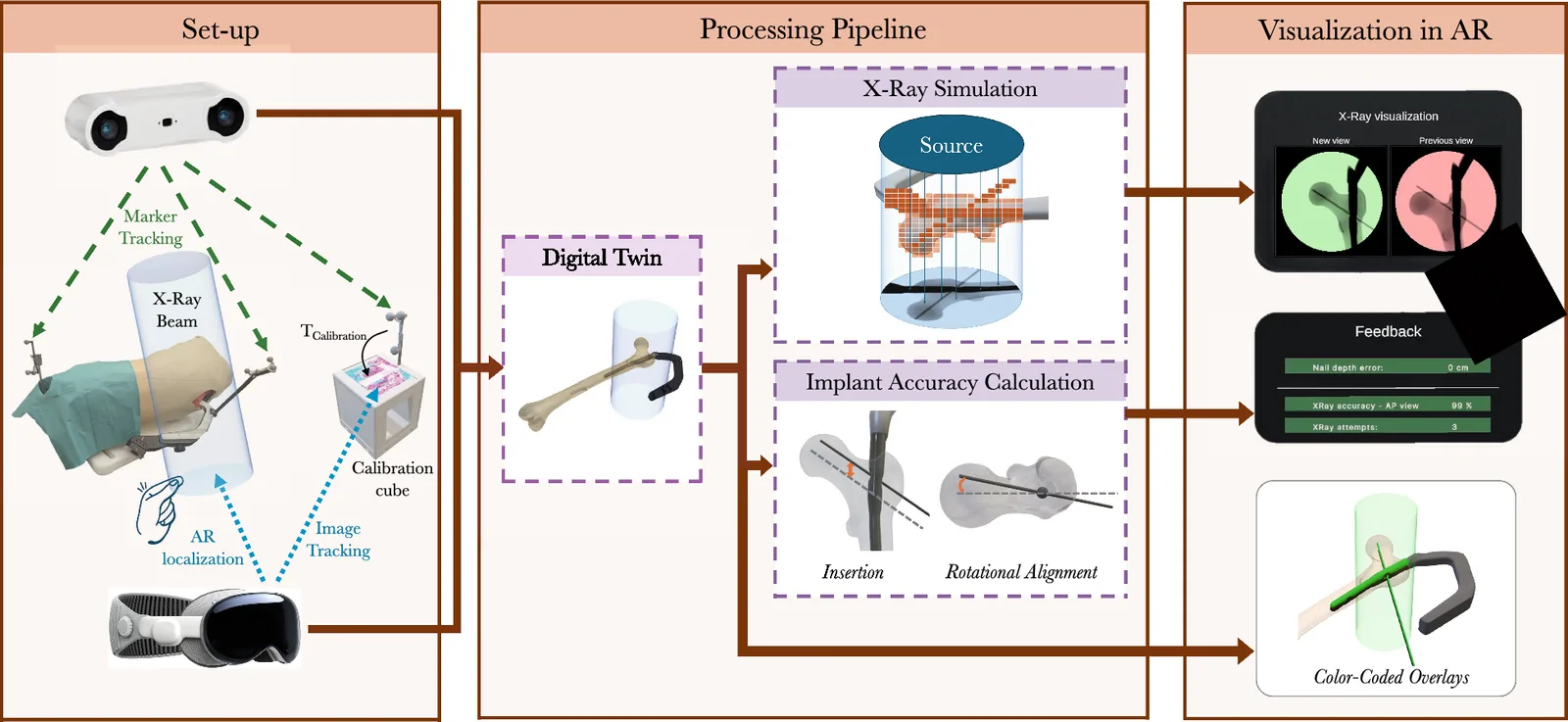
Data processing pipeline of the AR-enhanced femoral nailing simulator. Calibration between optical and image-based tracking ensures spatial alignment between the physical setup and the AR environment. Tracking data are integrated into a digital twin to generate simulated fluoroscopic images and to calculate implant accuracy. The processed information is visualized in AR through simulated X-rays, quantitative feedback, and color-coded overlays that provide real-time procedural guidance

The AR application operates on a real-time data processing pipeline (Fig. 3), ensuring spatial consistency between virtual and physical components, while enabling simulated fluoroscopic imaging, implant accuracy assessment, and real-time guidance via AR overlays.

The femur model and aiming arm were tracked using an optical tracking system (FusionTrack 250, Atracsys LLC, Switzerland). Both components were equipped with 3D-printed adapters carrying reflective marker spheres. The tracking data were streamed in real time to a local workstation (11th Gen Intel® Core$^{\textrm{TM}}$ i7-1165G7) and integrated into a Unity-based digital twin operating at 120 Hz. Digital models of the femur and instruments were obtained using a 3D scanner (Shining 3D, China) and registered offline to the physical objects. For this purpose, a tracked pointer was used to sample surface points on each object, generating point clouds that were registered to the corresponding digital models using an iterative closest point (ICP) algorithm. The processed data from the digital twin were streamed to the AR application running on the Apple Vision Pro, which operated at 60 Hz.

To align the coordinate systems of the optical tracking setup and the headset’s spatial mapping environment, a onetime calibration was performed during application initialization. A rigid marker assembly combining a reflective marker cluster visible to the tracking cameras and an image marker detectable by the headset via Unity PolySpatial was simultaneously tracked by both systems. The corresponding 6-DoF poses were used to compute a rigid transformation between the optical tracking coordinate frame and the headset’s world-anchored coordinate system, ensuring spatial correspondence between virtual and physical elements.

Based on this spatial alignment, simulated fluoroscopic images were generated. After the user positioned the virtual cylinder representing the X-ray beam orientation, the aligned poses of the cylinder, femur, and nail were used to compute a voxel-based radiographic projection along the cylinder’s longitudinal axis. Each voxel was assigned a material-specific attenuation coefficient to simulate X-ray attenuation. The nail was modeled with high attenuation to represent its metallic composition, while the femur was assigned lower cortical and cancellous coefficients to reproduce realistic radiographic contrast. The resulting X-ray image was rendered and displayed in the AR environment.

### Study design

25 participants were recruited from the Department of Orthopaedic and Trauma Surgery at the Cantonal Hospital of Lucerne, including 6 experts (attending surgeons) and 19 non-experts (3 medical students and 16 residents). All participants provided written informed consent, and the study was conducted in accordance with institutional ethical standards.

**Fig. 4:**
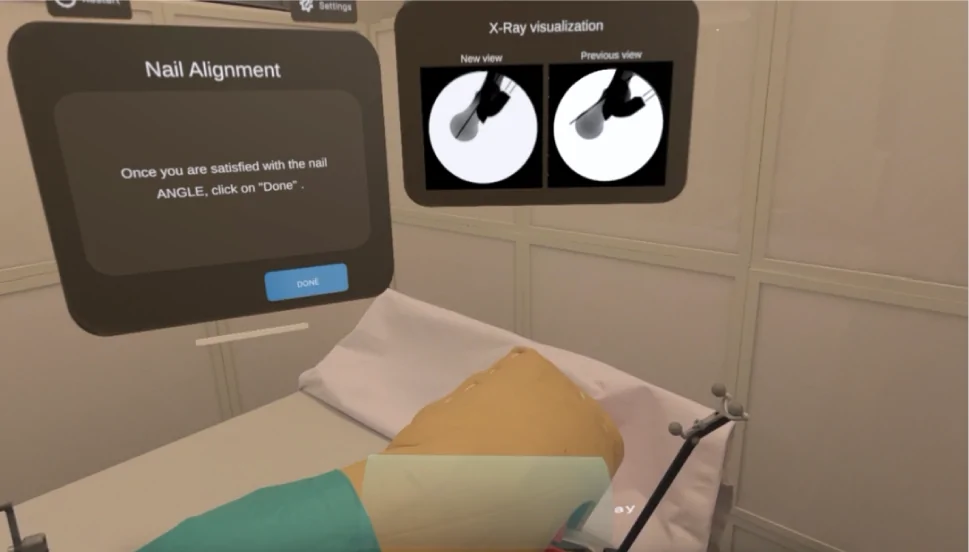
Simulation mode. During this unguided mode, all AR instructional features are disabled. Participants independently establish the correct fluoroscopic imaging orientation to determine the target nail insertion depth and rotational alignment

The participants followed an identical training sequence. After a brief introduction to the study objectives and the Apple Vision Pro, they received instruction in femoral nailing using the AR-enhanced simulator. They subsequently performed three consecutive trials in simulation mode without AR guidance (Fig. 4), practicing nail insertion and alignment independently. To mitigate memorization effects, different femur phantoms were used across sessions, introducing variability in entry-point position and mechanical resistance during nail insertion. Additionally, the femoral orientation was varied through internal–external rotation and abduction–adduction. After a minimum interval of one day, participants completed a retention session consisting of a single additional run in simulation mode to assess short-term skill retention. Performance was evaluated using two categories of outcome measures. All metrics were logged automatically during the simulation and retention sessions.

**Task Completion Time:** The time required to complete nail insertion and rotational alignment steps was recorded for every run. These data were used to quantify performance progression across repeated practice and to assess short-term retention.

**Procedural Quality:** (1) The number of simulated fluoroscopic images acquired: During insertion and alignment steps, the number of X-ray shots was recorded as a measure of radiographic navigation efficiency; (2) Implant placement accuracy: Accuracy of implant positioning was quantified at the end of each task. Insertion depth error was defined as the deviation between the achieved and ideal insertion depth (mm), and rotational alignment error as the deviation between the achieved and ideal implant orientation ($^{\circ }$).

### Statistical analysis

To evaluate within-subject changes in performance across repeated runs, paired comparisons were conducted using the two-sided Wilcoxon signed-rank test, as the distribution of paired differences could not be assumed to be normal. Learning effects were assessed through comparisons between the first and third simulation run, as well as between the first simulation run and the retention run. Statistical significance was defined as p<0.05. All analyses were performed in Python 3.10 (SciPy 1.15.3).

## Results

A total of 23 out of 25 participants were included in the analysis. Two participants had to be excluded due to data recording errors. The final cohort consisted of 7 female and 16 male participants, including 2 medical students, 15 residents, and 6 attending surgeons. The mean age was 31.9 years (SD = 7.2), with an average surgical experience of 5.8 years (SD = 6.8). The AR-guided instructional phase lasted on average 2.3 min for nail insertion and 1.0 min for rotational alignment before participants proceeded to the three unguided simulation runs. 18 (14 non-experts and 4 experts) of the 23 participants returned for the retention session after the initial training.

### Task completion time

**Fig. 5:**
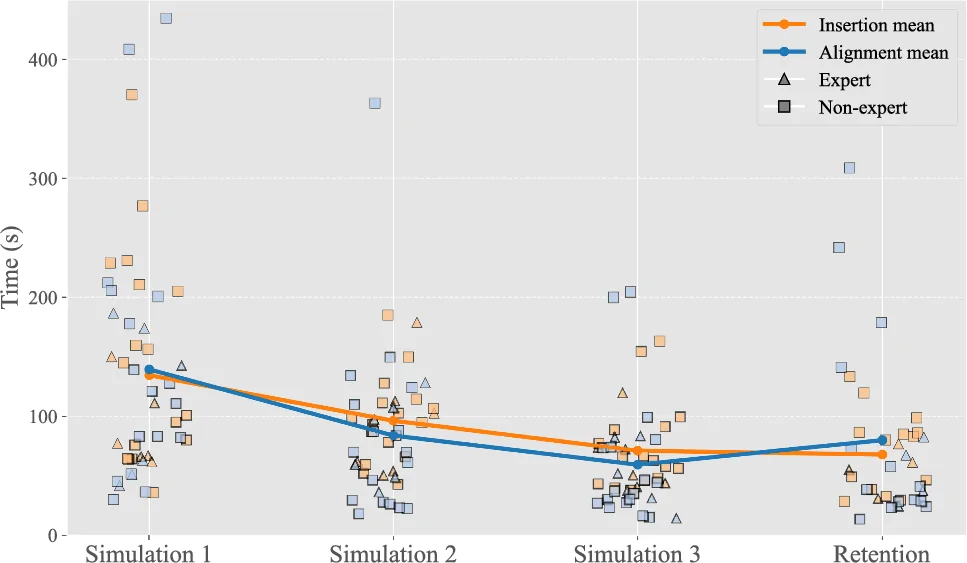
Task completion time per run for the insertion (orange) and alignment (blue) steps. Both tasks show reduced completion time across runs and stable performance in the retention session

Figure 5 shows task completion times for the insertion and alignment steps across all simulation runs and the retention run; within-subject changes were tested for statistical significance using paired two-sided Wilcoxon signed-rank tests.

For the nail insertion step, the average completion time decreased significantly from 134.7 s (median 101.0 s) in the first run to 71.2 s (median 62.9 s) in the third run (p<0.001). During the retention session, the average completion time remained stable at 68.0 s (median 69.1 s) and was still significantly lower than in the first run (p<0.001). Experts consistently performed faster than non-experts (Run 1: 89.1 s vs. 150.8 s; Run 2: 93.6 s vs. 97.4 s; Run 3: 67.0 s vs. 72.6 s; Retention: 56.2 s vs. 71.3 s), with the performance gap decreasing across runs.

A similar pattern was observed for the alignment step. The average completion time decreased significantly from 139.6 s (median 121.0 s) in the first run to 59.4 s (median 37.5 s) in the third run (p<0.001). In the retention session, the average completion time increased to 80.1 s (median 39.8 s) but remained significantly lower than in the first run (p=0.031). Experts completed the task faster (Run 1: 110.0 s vs. 150.0 s; Run 2: 79.8 s vs. 85.4 s; Run 3: 50.3 s vs. 62.6 s; Retention: 53.0 s vs. 87.8 s), with a diminishing difference between expert and non-expert performance over time.

### Procedural quality

**Fig. 6:**
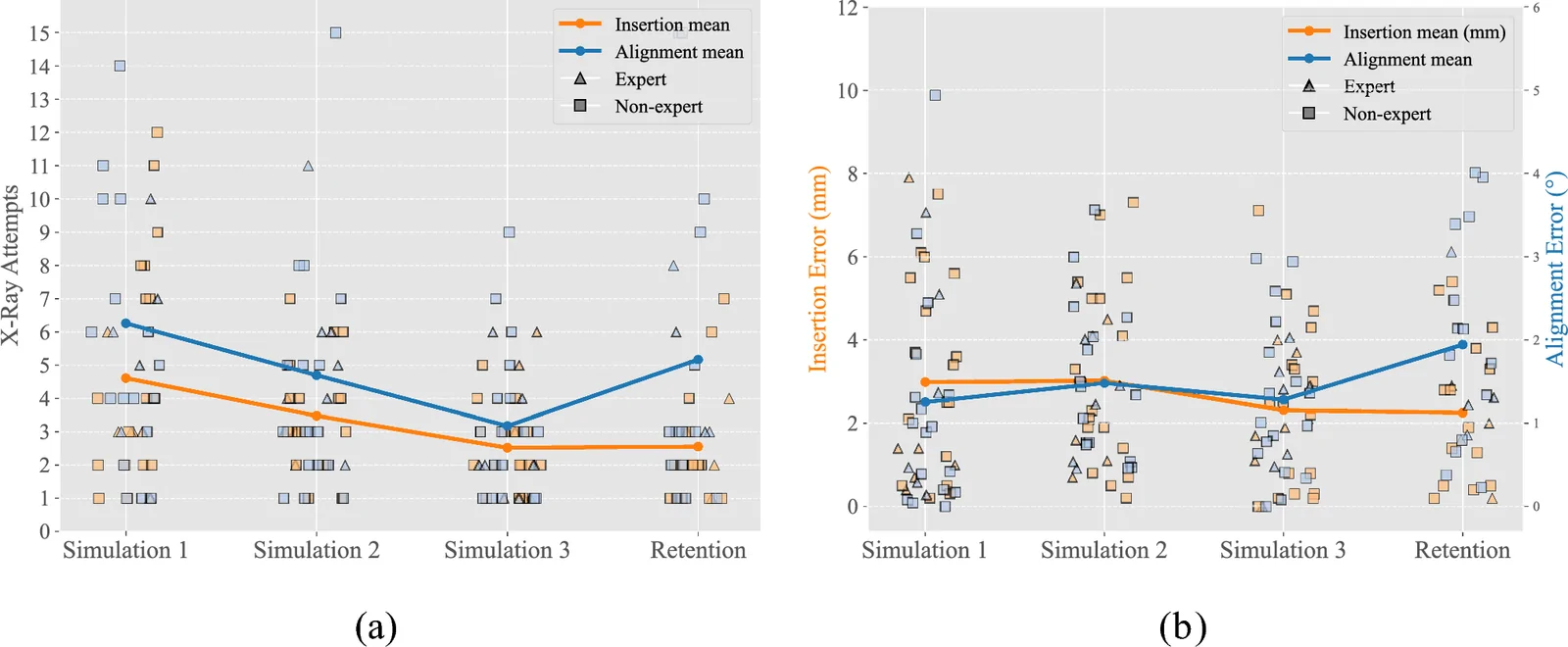
Results on procedural quality. **a** Number of fluoroscopic images acquired per run for insertion (orange) and alignment (blue). Training reduced fluoroscopic imaging usage, and performance remained largely stable in the retention session. **b** Implant placement accuracy per run for insertion depth (left axis) and alignment angle (right axis). Accuracy remained stable across the three training runs, with a slight increase in alignment error during the retention session

**Number of fluoroscopic images acquired:** Figure 6a shows the number of fluoroscopic images acquired during the insertion and alignment steps across all simulation runs and the retention run; within-subject changes were tested for statistical significance using paired two-sided Wilcoxon signed-rank tests.

For the insertion step, the average number of fluoroscopic images taken decreased significantly from 4.6 (median 3) in the first run to 2.5 (median 2) in the third run (p=0.0059). During the retention session, the average number of images taken remained similar at 2.6 (median 2); however, the difference compared to the first run was no longer significant (p=0.059). Experts required fewer images overall (Run 1: 3.2 vs. 5.1; Run 2: 3.3 vs. 3.5; Run 3: 2.8 vs. 2.4; Retention: 2.5 vs. 2.6), with differences becoming small after the second run.

For the alignment step, the average number of fluoroscopic images taken decreased significantly from 6.3 (median 5) in the first run to 3.2 (median 3) in the third run (p=0.0046). During the retention session, the average number increased to 5.2 (median 3), and was no longer significantly lower compared to the first run (p=0.442). Experts initially took more images in the first two runs (Run 1: 8.5 vs. 5.5; Run 2: 5.7 vs. 4.4), but from the third run onward, expert and non-expert performance converged (Run 3: 2.8 vs. 3.3; Retention: 5.0 vs. 5.2).

**Implant placement accuracy:** Figure 6b shows implant placement accuracy for the insertion and alignment steps across all simulation runs and the retention run; within-subject changes were tested for statistical significance using paired two-sided Wilcoxon signed-rank tests.

For the insertion step, the average deviation from the optimal implant depth decreased from 3.0 mm (median 2.5 mm) in the first run to 2.3 mm (median 2.3 mm) in the third run; however, the reduction was not significant (p=0.36). During the retention session, the average deviation remained similar at 2.2 mm (median 2.0 mm) and did still not differ significantly from the first run (p=0.12). Experts showed slightly greater precision (Run 1: 2.1 mm vs. 3.3 mm; Run 2: 2.5 mm vs. 3.2 mm; Run 3: 2.1 mm vs. 2.4 mm; Retention: 1.7 mm vs. 2.4 mm).

For the alignment step, the average angular deviation from the optimal implant orientation remained approximately 1.3$^{\circ }$ between the first (median 1.0$^{\circ }$) and the third run (median 1.4$^{\circ }$), with no significant change (p=0.73). During the retention session, the average deviation increased to 1.9$^{\circ }$ (median 1.8$^{\circ }$), but still did not differ significantly from the first run (p=0.24). No meaningful differences were observed between expert and non-expert groups (Run 1: 1.4$^{\circ }$ vs. 1.2$^{\circ }$; Run 2: 1.4$^{\circ }$ vs. 1.5$^{\circ }$; Run 3: 1.3$^{\circ }$ vs. 1.3$^{\circ }$; Retention: 1.6$^{\circ }$ vs. 2.0$^{\circ }$).

## Discussion

The present study evaluated an AR-enhanced physical simulator for femoral nailing, combining realistic haptic interaction with digital procedural guidance. The results demonstrate clear learning effects in time on task and X-ray handling, while implant placement accuracy remained stable across sessions.

The reduction in task completion time across the three simulation runs indicates procedural learning, which was retained in the follow-up session. Experts consistently performed faster than non-experts, suggesting that the simulator effectively trains skills relevant to real surgical performance rather than improving familiarity with the AR application itself. The decreasing number of simulated X-ray acquisitions reflects improved spatial understanding and radiographic planning, suggesting that repeated simulator use may help reduce intraoperative radiation exposure for patients and surgical staff, which remains a challenge in image-guided orthopedic surgery [8, 21].

In contrast, implant placement accuracy showed no significant improvement across runs. This likely reflects a ceiling effect, as participants had already achieved clinically acceptable precision after the guided instruction phase. The instruction emphasized reaching a defined accuracy range rather than minimizing residual error, leading participants to focus on efficiency once satisfactory placement was achieved. Consistent with the speed–accuracy trade-off principle described by Fitts et al. [22], participants appeared to shift their attention from precision toward procedural efficiency—completing tasks faster and with fewer simulated X-ray acquisitions—as they gained confidence in the procedure.

Overall, the findings highlight the potential of AR as a tool to enhance physical simulation environments. AR enables the integration of simulated fluoroscopic imaging, which allows users to practice radiographic navigation and three-dimensional spatial reasoning in a completely radiation-free and cost-efficient environment, without the need for additional physical imaging equipment. In addition, AR can facilitate the learning of minimally invasive procedures by visualizing otherwise hidden anatomy during training. This feature, used in the simulator, helps beginners understand the spatial relationships behind their actions and lowers the entry barrier to complex image-guided procedures.

A similar hybrid simulator presented by Wu et al. [23] has likewise demonstrated the promise of AR for improving procedural understanding and user engagement in surgical training. By combining real surgical instruments with AR-based visualization, the present system enables authentic tactile feedback while providing procedural support through immersive digital guidance. This approach bridges the strengths of both virtual and physical training modalities, supporting self-directed practice without compromising realism.

While the results are encouraging, this study was limited by the restricted scope of the simulated procedure, which included only two key steps rather than a complete surgical workflow. Future work could therefore focus on incorporating extended procedural scenarios. Long-term retention beyond the short follow-up interval was also not evaluated and should be addressed in longitudinal studies. In addition, no transfer study was conducted to assess how the acquired skills translate to real operating room performance. Clinical transfer could be evaluated in future work by comparing the operative performance of an AR-simulator-trained group against a control group of conventionally trained trainees, using metrics such as procedure time and fluoroscopic image count in real femoral nailing procedures. Such studies would further validate the educational value of the proposed AR-enhanced simulator.

## Conclusion

This study presented an AR-enhanced physical simulator for femoral nailing that combines realistic haptic interaction with digital procedural guidance. The system was developed to train fluoroscopic imaging orientation and implant placement within a realistic physical environment.

The results demonstrated clear learning effects in task efficiency and radiographic handling, indicating that the simulator effectively supports the acquisition of practical surgical skills. AR therefore represents a valuable extension to conventional physical simulators, enabling realistic yet self-directed training without the need for an instructor. These findings provide further evidence for the role of AR in surgical training and highlight its potential to enhance accessibility and realism in skill acquisition across a wide range of procedures, marking a further step forward in the ongoing shift toward simulation-based surgical training.
